# Soluble urokinase-type plasminogen activator receptor (suPAR) is a risk indicator for eGFR loss in kidney transplant recipients

**DOI:** 10.1038/s41598-021-83333-7

**Published:** 2021-02-12

**Authors:** Ulrich Jehn, Katharina Schütte-Nütgen, Ute Henke, Hermann Pavenstädt, Barbara Suwelack, Stefan Reuter

**Affiliations:** Division of General Internal Medicine, Nephrology and Rheumatology, Department of Medicine D, University Hospital of Muenster, 48149 Muenster, Germany

**Keywords:** Biomarkers, Medical research, Nephrology

## Abstract

The prognostic significance of suPAR in various kidney diseases has recently been demonstrated. Its role in transplantation-specific outcomes is still largely unknown. Therefore, we prospectively investigated the prognostic relevance of suPAR in patients before and one year after kidney transplantation (KTx). We included 100 patients who had received a kidney transplantation between 2013 and 2015. The plasma concentration of suPAR was measured by ELISA assay. In recipients of living donations (LD), pre-transplant suPAR levels were significantly lower than those of recipients of deceased donations (DD). After KTx, suPAR levels significantly declined in LD and DD recipients, without a detectable difference between both groups any more. Higher suPAR levels in recipients one year after KTx were associated with a more severe eGFR loss in the following three years in multivariable cox-regression (n = 82, p = 0.021). suPAR-levels above 6212 pg/ml one year after KTx are associated with eGFR loss > 30%, which occurred almost twice as fast as in patients with suPAR ≤ 6212 pg/ml (p < 0.001). Hence, suPAR level at one year mark might be a risk indicator of increased eGFR loss.

## Introduction

The soluble urokinase-type plasminogen activator receptor (suPAR) and its membrane-bound form uPAR are signaling proteins of the Ly6/alpha-neurotoxin family. They are secreted by immature myeloid cells, neutrophils, endothelial cells, smooth muscle cells, and podocytes, amongst others^1,2^. Notably, neutrophils can also serve as a major suPAR source in the bloodstream, at least in inflammatory conditions, when uPAR sheds from the neutrophil surface^3^. Both circulating and membrane-bound forms of suPAR are directly linked to cell adhesion and migration through binding integrins^2^. In the kidney, suPAR binds to and activates αvβ3 integrin on the podocyte membrane. Thereby, it contributes to podocyte foot process effacement and glomerular barrier function disruption, resulting in proteinuria^4,5^. Interestingly, it was found that uPAR-deficient mice are protected from lipopolysaccharide (LPS)-induced injury of the kidney filtration barrier. However, this protection can be reverted by the constitutive expression of αvβ3 integrin^5^.

An experimental study by Hahm et al. identified bone marrow (BM)-derived immature myeloid cells as a significant source of suPAR in LPS-treated mice. Knockout mice with uPAR deficiency were irradiated and reconstituted by BM cells of uPAR wild-type mice. These chimeric mice that selectively express uPAR within hematopoietic cells showed elevated suPAR levels upon stimulation with LPS, both in the blood and urine, as well as proteinuria^1^.

The expression of uPAR in the kidney and the concentrations of suPAR in the blood are consistently elevated in patients with kidney diseases, especially with focal segmental glomerulosclerosis (FSGS) and diabetic nephropathy^6–8^. In patients experiencing autosomal dominant polycystic kidney disease (ADPKD), higher suPAR levels indicate unfavorable disease courses, even though this type of kidney disease is not caused by podocyte injury^9^. In congruence, Hayek et al. also showed that elevated plasma levels of suPAR indicate emerging chronic kidney disease (CKD) in persons with normal kidney functions at the baseline^10^. The association of suPAR with outcome measures has recently been shown to persist in hemodialysis patients^11,12^.

The elevation of suPAR is not solely associated with kidney diseases, but was also linked to inflammatory and diverse pathologic conditions such as rheumatologic diseases^13,14^, acute respiratory distress syndrome (ARDS)^15^, or different types of cancer^16^.

It is still unclear whether suPAR has a pathophysiologic or prognostic role in kidney transplant (KTx) patients. At present, only a few KTx studies have tried to clarify its biomarker value for recurrent FSGS after KTx^17–19^. Regarding contrary results of different studies, Winnicki et al. showed that suPAR can serve as valuable biomarker for FSGS, but as a pitfall values must be interpreted in the context of population and test methods used considering test-specific cut-offs^17^. However, its role in kidney function or transplant specific outcomes needs further clarification. Therefore, we herein first prospectively investigated the prognostic relevance of suPAR in patients immediately before and one year after KTx and the influence of KTx on its values.

## Materials and methods

### Study population

In this study, we prospectively included 100 consented patients (age ≥ 18 years) in the final analysis who had received a kidney transplant at our transplant center between April 2013 and October 2015 and were able to provide both serum samples (at KTx, and 1-year after KTx) for suPAR analysis. We originally enrolled a total of 160 patients, but had to exclude 60 patients who did not present to our outpatient clinic for 1-year follow-up, withdrew their consent, changed the transplant center, died (n = 7) or lost their graft (n = 2) in the first year after KTx. Mean follow-up time was 4.35 ± 1.02 years, median follow-up time 4.35 years (IQR 3.80–5.35 years), respectively.

suPAR was measured in two different blood samples. The first sample was collected within 24 h before KTx, and the second was collected during a routine 1-year patient visit to our outpatient clinic. The initial immunosuppressive regimen consisted of basiliximab, tacrolimus (target trough 6–12 ng/mL), mycophenolate mofetil, and prednisolone. Anti-thymocyte globulin was administered to re-transplanted or highly immunized patients (PRA > 85%). AB0-incompatible patients received rituximab four weeks before KTx. Three patients with atypical hemolytic uremic syndrome (aHUS) as an underlying disease were given eculizumab in addition to basiliximab. Oral CMV-prophylaxis with valganciclovir was administered for 100 days in R + and 200 days in the D + /R−constellation.

### ELISA

Plasma concentrations of suPAR were measured using the Quantikine Human uPAR ELISA assay (R&D Systems, Minneapolis, MN, USA) according to the manufacturer’s instructions. The assay range is 62.5–4000 pg/ml, with a sensitivity of 33 pg/ml to suPAR. The samples above the concentration limit of the test were re-measured after tenfold dilution in Calibrator Diluent RD 6–10 reagent according to the manufacturer’s directions. Blood samples were collected after gaining informed consent at hospital admission within 24 h prior to KTx. The baseline and one-year samples were collected from each patient and immediately sent to the research core laboratory. The serum was obtained by centrifugation for 10 min at 2000 *g* using a refrigerated centrifuge, transferred into clean polypropylene tubes, and stored at − 80 °C until time of assay.

Patient characteristics were taken from the hospital’s electronic patient records. The data of all the participating patients was made anonymous prior to conducting an analysis. Moreover, written informed consent was obtained from all the participants. All experiments were performed in accordance with the current transplantation guidelines and the declarations of Istanbul and Helsinki. This study was approved by the local ethics committee, Ethik Kommission der Ärztekammer Westfalen-Lippe und der Medizinischen Fakultät der Westfälischen Wilhelms-Universität (No. 2013-364-f-S and No. 2019-109-f-S).

### Outcome measures

The main outcome measures involved renal function (eGFR calculated using the CKD-EPI equation^20^ and urine-protein/creatinine ratio (UPCR)) at years one to four after KTx. Other outcome parameters were patient and overall graft survival. Patient survival was defined as the time from KTx to death (due to any cause). When patients were lost to follow-up, the time from KTx to a patient's last contact was recorded. They were indicated as “alive”. Overall graft survival was defined as the time from KTx to death (from any cause), graft failure, or the last contact, whichever occurred first. Re-initiation of dialysis or re-transplantation was considered as graft failure. Recipients after LD and DD were considered separately^21^.

Patients were subjected to kidney biopsy in case of increased creatinine levels (≥ 0.3 mg/dL) and/or a significant increase in proteinuria. The kidney biopsies were evaluated by two independent pathologists. The whole blood was analyzed for creatinine (enzymatic assay; Creatinine-Pap, Roche Diagnostics, Mannheim, Germany). Organ rejections were diagnosed as per the BANFF classification^22^.

### Statistical analysis

Data was analyzed using IBM SPSS Statistics 26 (IBM Corp., Armonk, New York, USA). Normally distributed continuous variables have been presented as mean ± standard deviation (SD), and non-normally distributed continuous variables were presented as median and 1st and 3rd quartiles (interquartile range, IQR). Absolute and relative frequencies have been provided for categorical variables^21^.

Pairs of independent groups were compared using the Student’s t-test for normally distributed data, the Mann–Whitney U test for non-normal data, and the Fisher’s exact test or Chi-Quadrat-Test for categorical variables. To compare the paired data, we used the Wilcoxon test for continuous variables and the McNemar test for categorical variables.

The cumulative probability of developing eGFR loss > 30% in our KTx cohort was calculated using the Kaplan–Meier analysis, and the curves were compared using the log-rank test. A cut-off for suPAR after one year for patients at higher risk for eGFR-loss was identified by calculating Youden-indices based on a ROC-analysis.

To evaluate independent risk factors for the onset of eGFR loss > 30%, we performed multivariable Cox-regression analyses.

## Results

Demographic and clinical characteristics have been presented in Table 1. 56 patients (56%) received living donations (LD), and 44 (44%) had deceased donations (DD). 17 (30.4%) of the LD were AB0-incompatible transplantations. 64 recipients (64%), the majority, were male. The mean age of the recipients at the time of KTx was 49.3 years (SD = 15.62).

**Table 1:** Baseline characteristics of the recipients.

	All (n = 100)	Living (n = 56)	Deceased (n = 44)	p-value
Age (years, mean ± SD)	49.31 (15.62)	42.76 (15.06)	57.65 (12.02)	0.000^a^
Male, n (%)	64 (64)	35 (62.5)	29 (65.9)	0.728^a^
Diagnosis of ESRD, n (%)				0.498^b^
Hypertension	13 (13)	7 (12.5)	6 (13.6)	
Diabetes	3 (3)	0 (0)	3 (6.8)	
Polycystic kidney disease	13 (13)	6 (10.7)	7 (15.9)	
Obstructive nephropathy	4 (4)	3 (5.3)	1 (2.3)	
Glomerulonephritis	33 (33)	21 (37.5)	12 (27.3)	
FSGS	7 (7)	5 (8.9)	2 (4.5)	
Interstitial nephritis	3 (3)	1 (1,8)	2 (4.5)	
Vasculitis	2 (2)	2 (3.6)	0 (0)	
Other	22 (22)	11 (19.6)	11 (25)	
Mode of dialysis, n (%)				0.009^b^
Hemodialysis	71 (71)	40 (71.4)	31 (70.4)	
Peritoneal dialysis	12 (12)	6 (10.7)	6 (13.6)	
Both	9 (9)	2 (3.6)	7 (16)	
Preemptive donation	8 (8)	8 (14.3)	0 (0)	
Time on dialysis (months, mean (1^st^ & 3^rd^ quartile))	44.9 (11.3, 79.2)	19.7 (0.9, 26.2)	77.5 (41.6, 102.2)	0.000^c^
Immunized	38 (38)	21 (37.5)	17 (39)	0.907^d^
European senior program (ESP)	14 (14)	0 (0)	14 (32)	0.000^d^
≥ 1 prior kidney transplantat, n (%)	14 (14)	8 (14.3)	6 (13.6)	0.926^d^
Current PRA, n (%)				0.567^b^
0–20%	73 (73)	41 (73.2)	32 (73)	
> 20%	27 (73)	15 (26.8)	12 (27.3)	
Induction, n (%)				0.000^b^
Basiliximab	75 (75)	38 (67.8)	37 (84.1)	
Thymoglobuline	5 (5)	0 (0)	5 (11.3)	
Basiliximab + Thymoglobuline	1 (1)	0 (0)	1 (2.3)	
Rituximab + Thymoglobuline	1 (1)	1 (1.8)	0 (0)	
Rituximab + Basiliximab	16 (16)	16 (28.6)	0 (0)	
Eculizumab + Basiliximab	2 (2)	1 (2.6)	1 (2.3)	
Cold ischemia time (hours, mean ± SD)	5.77 (7.8)	2.47 (0.64)	9.96 (3.3)	0.000^a^
Warm ischemia time (min, mean ± SD)	34.3 (4.35)	33.46 (8.03)	35.4 (7.36)	0.211^a^
AB0i	17 (17)	17 (30.4%)	0 (0)	0.000^b^
eGFR 365 days (ml/min/1.73 m^2^, mean ± SD)	57.32 (20.06)	60.01 (20.25)	53.9 (19.5)	0.131^a^

Demographic characteristics of the study population. The results have been presented as mean ± standard deviation or median and 1^st^ and 3^rd^ quartile, respectively, or as absolute and relative frequencies.

*ESRD* end-stage renal disease, *FSGS* focal segmental glomerulosclerosis, *ESP* European Senior Program, *HLA* human leukocyte antigen, *PRA* panel reactive antibodies, *eGFR* estimated glomerular filtration rate, *SD* standard deviation.

^a^Mann–Whitney U test.

^b^Fisher’s exact test.

^c^Kruskal–Wallis test.

Patients’ outcome data have been presented in Table 2.

**Table 2:** Outcomes of the recipients.

	All (n = 100)	Living (n = 56)	Deceased (n = 44)	p-value
suPAR pre KTx median (1st & 3rd quartile)	8095 (5818, 11,192)	6,839 (4.887, 10.183)	9,117 (6,891, 12,211)	**0.008**^**a**^
suPAR day 365 median (1st & 3rd quartile)	4376 (2757, 5612)	4332 (2,914, 5,567)	4413 (2575, 6049)	**0.879**^**a**^
eGFR day 365 (ml/min/1.73m^2^, mean ± SD)	57.3 ± 20.1	60.0 ± 20.3	53.9 ± 19.5	**0.075**^**a**^
eGFR day 720 (ml/min/1.73m^2^, mean ± SD)	54.7 ± 20.1	56.4 ± 18.8	52.6 ± 21.6	**0.165**^**a**^
eGFR day 1080 (ml/min/1.73m^2^, mean ± SD)	53.9 ± 17.6	55.5 ± 17.6	52.0 ± 17.7	**0.153**^**a**^
eGFR day 1440 (ml/min/1.73m^2^, mean ± SD)	51.8 ± 15.9	53.8 ± 14.3	49.5 ± 17.4	**0.287**^**a**^
UPCR day 365 (mg/g crea, mean ± SD)	284 ± 906	308 ± 1,124	101 ± 519	**0.884**^**a**^
UPCR day 720 (mg/g crea, mean ± SD)	165 ± 218	157 ± 190	174 ± 255	**0.597**^**a**^
UPCR day 1,080 (mg/g crea, mean ± SD)	163 ± 187	167 ± 212	158 ± 151	**0.726**^**a**^
UPCR day 1440 (mg/g crea, mean ± SD)	169 ± 201	176 ± 240	162 ± 156	**0.885**^**a**^
eGFR-loss > 30% from year one, n (%)	20 (20%)	14 (25%)	6 (13.6%)	**0.210**^**a**^
**Rejection, n (%)**	46 (46%)	29 (51.8%)	17 (38.6%)	**0.069**^**b**^
Antibody-mediated rejection	14 (14%)	10 (17.9%)	4 (9.1%)	
T-cellular rejection	5 (5%)	4 (7.1)	1 (2.3%)	
Combined rejection	4 (4%)	2 (3.6)	2 (4.5%)	
T-cellular borderline rejection	22 (22%)	12 (21.4)	10 (22.7%)	
CMV viremia, n (%)	21 (21%)	5 (8.9%)	16 (36.4%)	**0.001**^**b**^
BKPyV viremia, n (%)	20 (20%)	7 (12.5%)	13 (29.5%)	**0.043**^**b**^
NODAT, n (%)	20 (20%)	7 (12.5%)	13 (29.5%)	**0.045**^**b**^

*suPAR* Soluble urokinase-type plasminogen activator receptor, *eGFR* estimated glomerular filtration rate, calculated using CKD-EPI formula, *UPCR* urine protein creatinine ratio, *CMV* cytomegalovirus, *BKPyV* BK-Polyomavirus, *NODAT* New-onset diabetes after transplantation, *crea* creatinine.

^a^Mann–Whitney U test.

^b^Fisher’s exact test.

**Figure 1:**
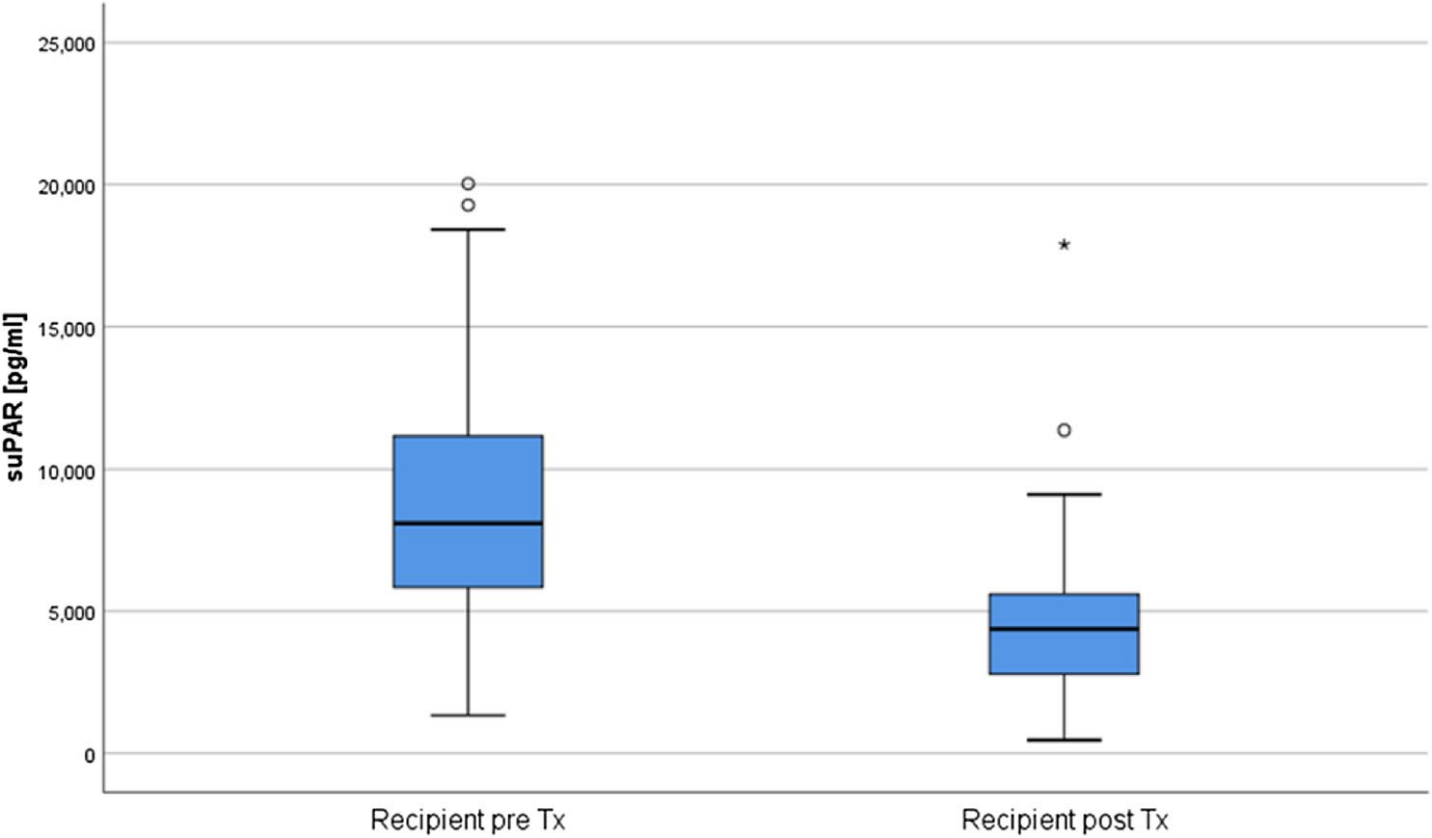
Recipients had significantly lower suPAR levels one year after KTx compared to suPAR levels before KTx.

In patients who received LD, pre-transplant suPAR levels were significantly lower compared to those receiving DD (suPAR 6839 (4887, 10,183) pg/ml, vs. 9117 (6891, 12,211) pg/ml, p = 0.008). suPAR levels significantly declined in LD and DD recipients after KTx (8095 (5818, 11,192) pg/ml before KTx vs. 4376 (2757, 5612) pg/ml one year after KTx; Fig. 1), (n = 100, p < 0.001).

**Figure 2:**
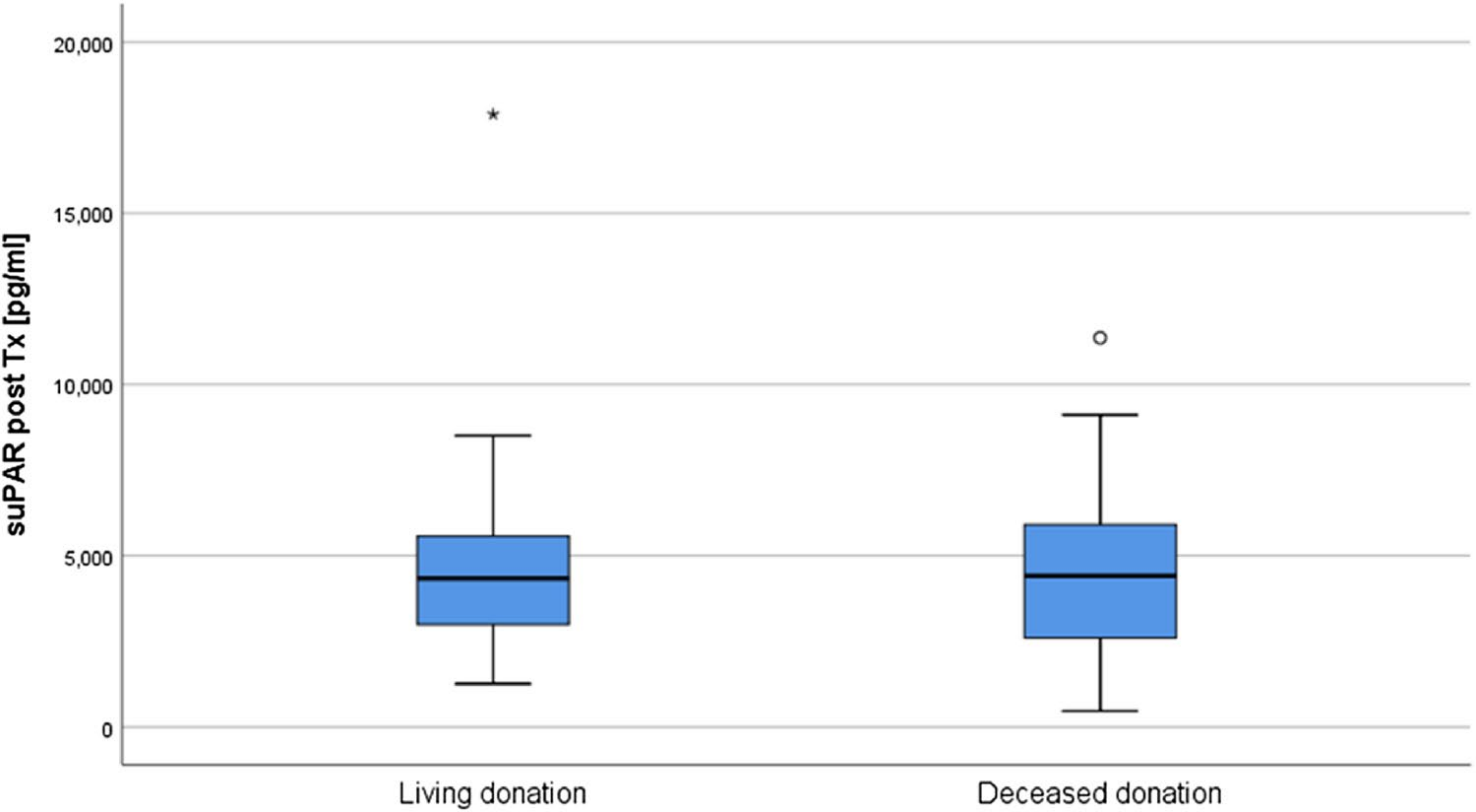
Recipients’ suPAR levels after LD and DD aligned 1 year after KTx.

In detail, the suPAR levels of recipients aligned without any detectable difference between those who had LD and DD (LD 4332 (2914, 5567) pg/ml vs. DD 4413 (2575, 6049) pg/ml, n = 100, p = 0.879, Fig. 2).

In parallel, it was found that kidney function improved one year after KTx to a mean eGFR of 57.3 ± 20.1 ml/min/1.73 m^2^ (Table 1). Although LD recipients showed a tendency toward a higher eGFR compared to DD recipients (60.0 ± 20.3 ml/min/1.73 m^2^ vs. 53.9 ± 19.5 ml/min/1.73 m^2^, p = 0.075), the difference was not statistically significant.

Dialysis vintage and cold ischemic time were significantly shorter in LD than DD recipients (19.7 months vs. 77.5 months, p < 0.001; 2.47 h vs. 9.96 h, p < 0.001).

**Figure 3:**
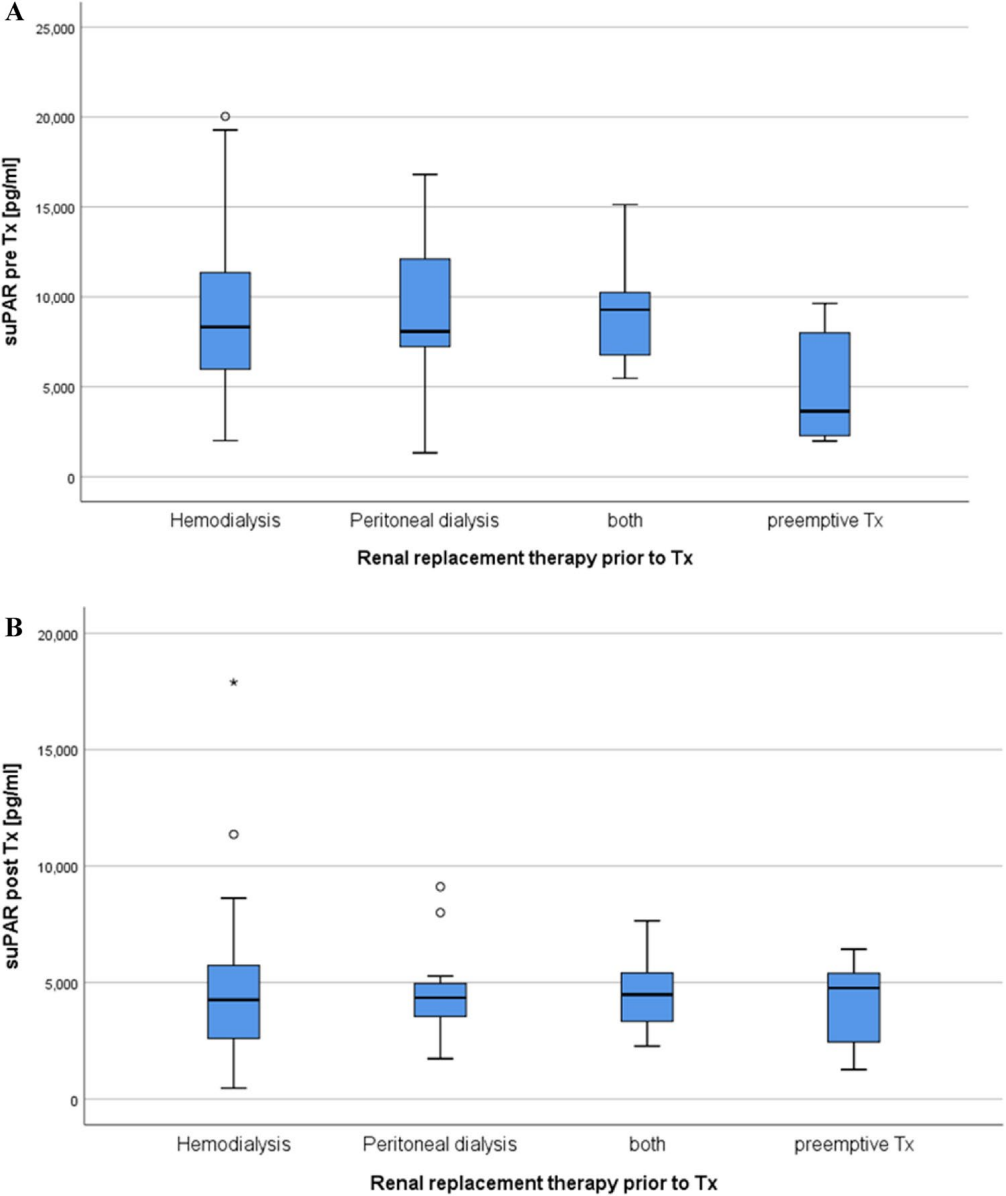
(**A**,**B**) The suPAR levels in recipients receiving preemptive donations are significantly lower compared to the patients receiving hemo- or peritoneal dialysis. One year after transplantation, the suPAR levels of both cases were comparable.

Dialysis vintage tended to be associated with suPAR levels prior to KTx (n = 100, p = 0.067,). Upon a closer analysis, it was seen that preemptive recipients who never underwent dialysis had significantly lower suPAR levels (suPAR 5249 (2302, 7806) pg/ml, n = 8) compared to patients on any mode of dialysis before transplantation (suPAR 8392 (6011, 11,503) pg/ml, n = 92) (p = 0.006) (see Fig. 3A). The suPAR levels of patients on hemodialysis (suPAR 8322 pg/ml (5956, 11,475), n = 71) and patients treated with peritoneal dialysis (8075 (6936, 12,492) pg/ml, n = 12) were comparable (p = 0.928) (see Fig. 3A). One year after KTx, the suPAR levels of patients on any mode of dialysis prior to transplant compared with preemptively transplanted patients became equal (Fig. 3B).

**Figure 4:**
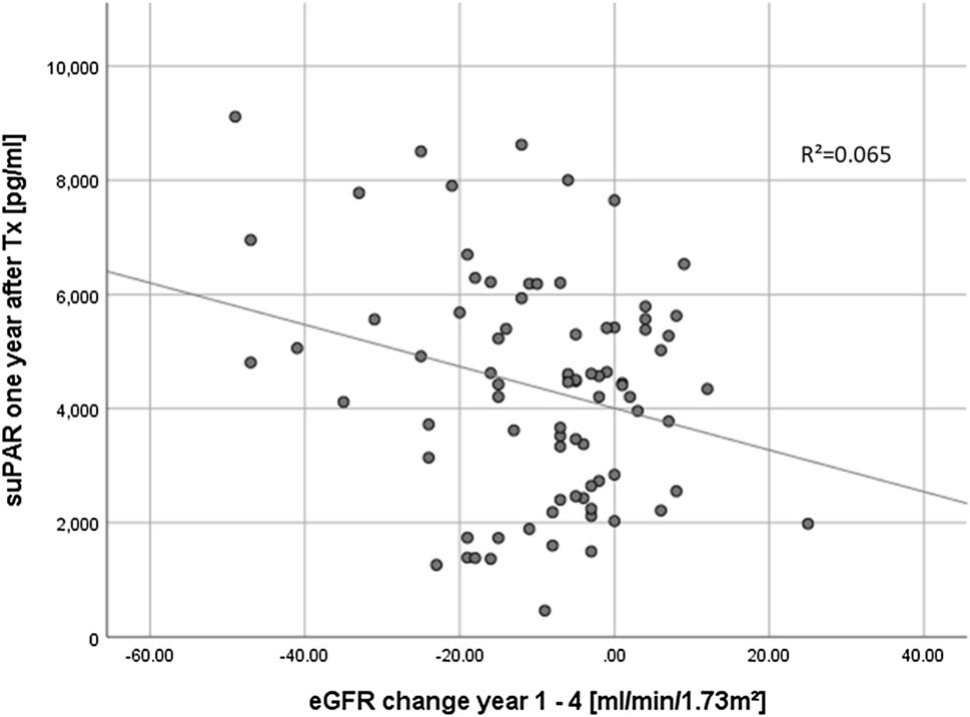
The correlation between the recipients’ suPAR levels at day 365 and eGFR decline in the following three years. Higher suPAR-levels preceded a higher eGFR-loss in the following three years after KTx.

The suPAR levels in patients one year after KTx were not correlated to the eGFR at the same time (p = 0.24, r = − 0.119). However, it was associated with the development of the eGFR between the second and fourth year after KTx. Higher suPAR levels one year after KTx could be associated with a higher eGFR-loss in the following three years (Fig. 4, n = 82 (18 patients lost of follow-up), p = 0.021, r = − 0.255).

In our study, a correlation between the suPAR levels and the incidence or the number of biopsy-proven allograft rejections could not be detected.

We found that only four patients experienced terminal graft failures by the time of the follow-up. Moreover, two patients died during the course of the study, and two patients died without losing their graft before. Due to these small number of events, we did not perform survival analyses with these endpoints.

**Figure 5:**
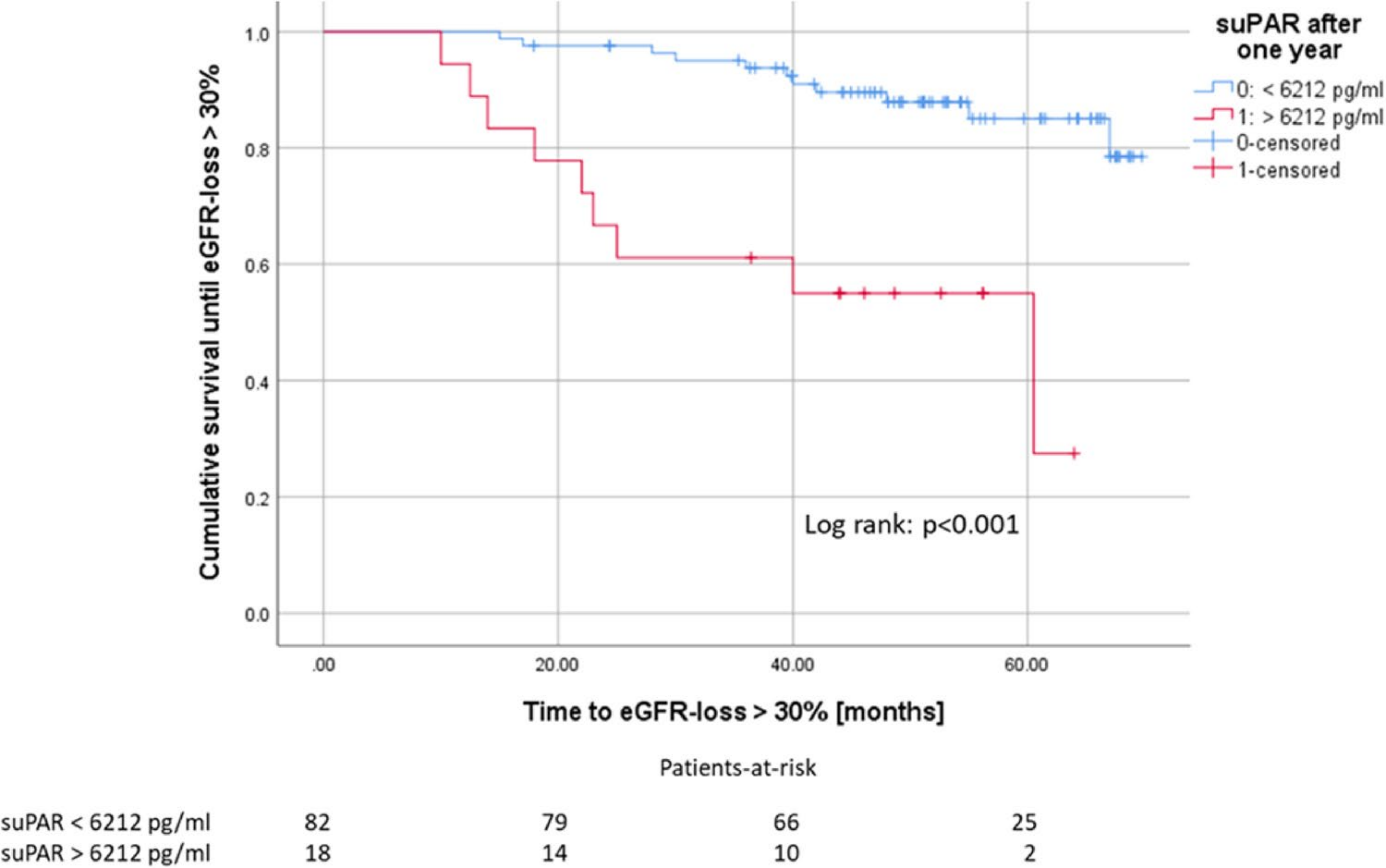
The suPAR-levels above 6212 pg/ml measured one year after KTx are significantly associated with an increased loss of allograft function.

Instead, we took a loss of renal function in terms of more than 30% of eGFR loss from year one as an endpoint^23,24^. eGFR-loss > 30% was stated, when it was constant for at least one month and did not increase subsequently. Our patient collective was divided into two groups based on a cut-off for suPAR below and above 6212 pg/ml by calculating the Youden-indices for a receiver operating characteristic analysis according to suPAR measured after one year. (n = 82 vs. n = 18). Patients with allograft loss were defined as eGFR-loss > 30%, patients who died with unimpaired allograft function were handled as negative for eGFR-loss > 30%. The Kaplan–Meier analysis and Log-rank test showed a significantly reduced time to eGFR-loss > 30% for patients with suPAR levels above 6212 pg/ml (33.5 vs. 61.9 months, Fig. 5).

### The suPAR levels after one year as an independent risk factor for eGFR-loss > 30% subsequently

To evaluate whether suPAR after one year is independently associated with accelerated eGFR-loss survival, apart from other known risk factors, we performed a multivariable Cox-regression analysis that included several known risk factors causing inferior allograft survival (Table 3).

**Table 3:** Cox-regression analysis for determining risk factors associated with *eGFR loss* > *30%*

Variable	Hazard Ratio	95% CI	P-value
Age at KTx	1.134	1.023–1.058	0.017
Previous KTx	39.639	3.912–401.605	0.002
CMV-viremia	1.062	0.061–18.494	0.967
BKV-viremia	2.121	0.071–63.367	0.664
Postmortal donation	2.481	0.023–266.090	0.703
NODAT	0.919	0.067–12.537	0.949
Time on dialysis	1.010	0.982–1.038	0.502
Cold ischemia time	0.685	0.341–1.373	0.286
Delayed graft function	0.395	0.010–15.217	0.618
Acute rejection	4.057	0.708–23.259	0.116
eGFR day 365	1.016	0.970–1.064	0.507
UPCR day 365	1.000	1.000–1.001	0.277
hs-CRP day 365	1.472	0.843–2.571	0.174
BMI	0.907	0.695–1.182	0.469
HLA-mismatches	0.379	0.141–1.018	0.054
Number of antihypertensive agents day 365	–	–	0.480
Mode of dialysis	–	–	0.961
Induction therapy	–	–	0.108
suPAR pre KTx	1.000	1.000–1.000	0.370
suPAR day 365	1.001	1.000–1.001	< 0.001

*KTx kidney transplantation, *CMV* cytomegalovirus, *BKV* BK-polyomavirus, *NODAT* new onset of diabetes after transplantation, *eGFR* estimated glomerular filtration rate, *UPCR* urine protein/creatinine ratio, *hs-CRP* high sensitivity C-reactive protein, *BMI* body mass index, *HLA* human leukocyte antigen, *suPAR* soluble urokinase-type plasminogen activator receptor.

Besides the well-known risk factors for kidney allograft failure such as age at the time of KTx (p = 0.017, HR 1.134) and previous KTx (p = 0.02, HR 39.639), the Cox-regression analysis confirmed suPAR one year after transplantation as an independent risk factor for eGFR-loss > 30% (p < 0.001, HR 1.001).

## Discussion

suPAR levels are high in patients with chronic proteinuric and non-proteinuric kidney diseases and may predict disease courses^7,10^. A recent study by Hayek et al. provided evidence that suPAR may be directly involved in the pathogenesis of acute kidney injury. Higher preprocedural suPAR levels were shown to be associated with subsequent acute kidney injury after different medical procedures, e.g. coronary angiography. These findings were experimentally confirmed in a mouse model for acute kidney injury. Mechanistically, Hayek et al. described a sensitization of kidney proximal tubules by suPAR to injury through modulation of cellular bioenergetics and increased oxidative stress in a cell culture model, which was not found for podocytes^25^. However, Wei et al. described a mechanism for suPAR leading to podocyte foot process effacement through the activation of αvβ3 integrin in podocytes^5^, thereby acting as a driver of kidney injury. Moreover, Hahm et al. reported that BM-derived immature myeloid cells are responsible for the elevated pathological levels of suPAR in the case of LPS-treated mice with proteinuric kidney disease and thus, they described suPAR as a key contributor to glomerular dysfunction^1^.

In summary, whether suPAR acts as an originator of kidney injury, is produced and/or released as a consequence of (kidney) injury, or both can occur concurrently has not been completely elucidated yet^26^. Since data on suPAR and outcome after KTx apart from FSGS patients is still absent, we performed this prospective study.

Our data shows that after KTx suPAR levels improved significantly (Fig. 1), in parallel with an increase in renal function (Table 1). Notably, there were distinct differences between LD and DD recipients. The suPAR levels in DD recipients were significantly higher compared to those receiving LD. This could perhaps be related to the fact that eight (16%) of the LD were preemptive with significantly lower suPAR values compared to those on dialysis prior to transplantation (Fig. 3A). Moreover, their dialysis vintage was shorter, and these patients were younger than DD recipients (Table 1). Interestingly, dialysis vintage was significantly correlated to suPAR levels prior to transplantation (p = 0.013, r = 0.258). In congruence, among other factors, dialysis vintage as well as the age of the patient have recently been linked to the suPAR levels in dialysis patients^11,12^. Additionally, since suPAR is excreted by the kidneys, end-stage renal disease may lead to an accumulation in the serum^27^. After KTx, the suPAR levels of both LD and DD recipients decreased to a comparable level at the 1-year mark (Fig. 2).

Similarly, morbidity and mortality of DD recipients are usually higher which is, among others, related to longer dialysis vintage^28,29^. Notably, higher suPAR levels have been shown to translate into all-cause and both CVD and non-CVD mortality in hemodialysis patients^11,12^. Thus, one may only speculate that lower suPAR levels in preemptive LD might contribute to preferable outcomes of LD KTx^28^. However, our study sample was too small to allow us to draw such conclusions.

Serum suPAR levels can indicate CKD prior to measurable function loss^10^. As the suPAR levels decline after KTx and as the native recipient kidneys usually remain in-situ after transplantation, the pathologically altered kidneys seem to be ineligible as a relevant suPAR source (due to progressive loss of function); otherwise, suPAR could be excreted by the working graft after transplantation, hence, kidneys seemed to have cleared suPAR from the circulation, at least in healthy volunteers^30^. However, our data confirmed the prognostical value of suPAR to predict the decline in functionality of the allograft (Figs. 4 and 5).

We observed that the suPAR level one year after KTx is predictive of future graft function (eGFR) to some extent, and suPAR levels above a cut-off of 6212 pg/ml serve as a risk factor for a significantly accelerated decrease of eGFR. Notably, even after adjustment for several other known risk factors for accelerated loss of allograft function, suPAR levels remained significantly predictive for eGFR loss one year after KTx (see Table 3). This is in line with the observations made in CKD patients^9,10^. One should keep in mind that different suPAR tests from different manufacturers may result in different suPAR cut-off values despite high sensitivity and specificity, as recently reported by Winnicki et al.^17^.

Interestingly, there are interferences in the αvβ3 integrin pathway, which is induced by suPAR and calcineurin-mediated processes. These clacineurin-mediated processes are suppressed in KTx patients through the administration of calcineurin inhibitors (CNI) such as tacrolimus or cyclosporine. CNI exhibit antiproteinuric effects by stabilizing the actin cytoskeleton and stress fibers of podocytes through synaptopodin re-storage^31,32^. Furthermore, in lupus nephritis, a combination therapy of tacrolimus and mycophenolate mofetil (a standard of care after KTx)^33^, had an additive protective effect for the podocyte actin cytoskeleton by tacrolimus-induced synaptopodin-mediated activation of RhoA and mycophenolate mofetil-mediated VAV1 inhibition of Rac1^32^.

However, our study has limitations, as it is a single-center study and observational in nature. Further, for our study, we analyzed data of a relatively small cohort, which did not include data on non-renal causes for eGFR decline or proteinuria such as infections, CNI toxicity, or surgical problems. Another study limitation is that GFR was estimated using serum creatinine-based CKD-EPI formula instead of cystatin C-based calculation, which has some advantages, such as higher sensitivity and specificity, over creatinine measurement^34^.

In conclusion, on the one hand, our observations implicate that elevated suPAR is a consequence of kidney disease and chronical inflammation, as KTx drops suPAR levels significantly after resolving the state of end-stage renal disease. On the other hand, suPAR seems capable as an early marker for allograft dysfunction after KTx.

## Data Availability

The datasets generated during and/or analyzed during the current study are available from the corresponding author on reasonable request.
